# Sex-specific local life-history adaptation in surface- and cave-dwelling Atlantic mollies (*Poecilia mexicana*)

**DOI:** 10.1038/srep22968

**Published:** 2016-03-10

**Authors:** Rüdiger Riesch, David N. Reznick, Martin Plath, Ingo Schlupp

**Affiliations:** 1 School of Biological Sciences, Centre for Ecology, Evolution and Behaviour, Royal Holloway University of London, Egham, Surrey TW20 0EX, UK; 2 Department of Biology, University of California, Riverside, CA 92521, USA; 3 Northwest A&F University, College of Animal Science and Technology, Xinong Road 22, Yangling 712100, Shaanxi Province, P.R. China; 4 Department of Biology, University of Oklahoma, 730 Van Vleet Oval, Norman, OK 73019, USA

## Abstract

Cavefishes have long been used as model organisms showcasing adaptive diversification, but does adaptation to caves also facilitate the evolution of reproductive isolation from surface ancestors? We raised offspring of wild-caught surface- and cave-dwelling ecotypes of the neotropical fish *Poecilia mexicana* to sexual maturity in a 12-month common garden experiment. Fish were raised under one of two food regimes (high vs. low), and this was crossed with differences in lighting conditions (permanent darkness vs. 12:12 h light:dark cycle) in a 2 × 2 factorial design, allowing us to elucidate potential patterns of local adaptation in life histories. Our results reveal a pattern of sex-specific local life-history adaptation: Surface molly females had the highest fitness in the treatment best resembling their habitat of origin (high food and a light:dark cycle), and suffered from almost complete reproductive failure in darkness, while cave molly females were not similarly affected in any treatment. Males of both ecotypes, on the other hand, showed only weak evidence for local adaptation. Nonetheless, local life-history adaptation in females likely contributes to ecological diversification in this system and other cave animals, further supporting the role of local adaptation due to strong divergent selection as a major force in ecological speciation.

Unravelling the mechanisms underlying the origin of biodiversity is a pivotal goal in evolutionary biology. Different environments exact different selective pressures on the organisms inhabiting them, so that—all else being equal—divergent selection should result in each local population evolving traits that are beneficial in their native habitat irrespective of whether or not these traits might affect performance in another (foreign) habitat. Hence, traits that are beneficial in the native habitat might turn out to be maladaptive in a foreign habitat, a phenomenon known as local adaptation^1^,^2^,^3^. However, adaptive trait divergence can also facilitate reproductive isolation and ultimately speciation^4^,^5^, a phenomenon termed ‘isolation-by-adaptation’^6^. For example, pre-zygotic isolation between different locally adapted populations will arise when immigrants from foreign, ecologically divergent habitats are selected against^7^,^8^. This may occur by sexual selection, if poorly adapted individuals are discriminated against during mate choice^9^,^10^,^11^, or by natural selection, if immigrants have a reduced fitness (extrinsic reproductive isolation)^12^.

Kawecki and Ebert^1^ postulated that locally adapted populations should have higher fitness in their ‘home’ habitat compared to their fitness in an ‘away’ habitat (i.e., ‘home versus away’; see also)^4^. Moreover, within their home habitat, individuals from the native population should have a higher fitness than individuals from a population native to a different habitat (‘native versus foreign’). Local adaptation is only fully confirmed, the authors argue, when both criteria are fulfilled^1^.

Cave animals are increasingly utilized in studies on adaptive trait divergence, including morphology, behaviour, EvoDevo, and life histories (reviewed by)^13^. However, testing whether Kawecki and Ebert’s^1^ criteria for local adaptation are fulfilled is limited by these comparisons being made across species boundaries^13^. In the present study, we investigated the potential for local adaptation facilitating reproductive isolation in surface versus cave populations of the Atlantic molly (*Poecilia mexicana*, Poeciliidae). This system is an example in which cave fishes co-occur with their closely related surface-dwelling relatives within the same river system^14^,^15^, providing an excellent opportunity to investigate how adaptation to a cave environment affects life histories and, indirectly, reproductive isolation between surface and cave populations.

Populations of *P. mexicana* inhabit a hydrogen sulphide-containing cave (Cueva del Azufre; henceforth CdA) as well as a variety of surface habitats in the Río Grijalva/Usumacinta drainage within a few kilometres of each other^16^,^17^,^18^,^19^. Hydrogen sulphide (H_2_S), presumably of volcanic origin^20^, reaches concentrations of >300 μM in CdA^18^,^21^,^22^. H_2_S is toxic to most metazoans because it interferes with mitochondrial respiration and blood oxygen transport, while simultaneously leading to extreme hypoxia of the water^23^.

Several factors other than darkness and H_2_S toxicity could serve as sources of ecologically-based divergent selection between surface and cave dwelling mollies. For example, cave mollies occur in much higher population densities than surface mollies^24^. Because of the hypoxia associated with H_2_S, cave mollies spend most of their time in the top-most layers of the water column to engage in aquatic surface respiration^25^,^26^. The need for aquatic surface respiration renders CdA a resource-limited habitat for cave mollies despite the presence of chemoautotrophic bacterial primary production^27^, as they must venture away from the water surface to graze on the energy-rich bacterial mats^26^,^28^. Examination of field-caught individuals revealed that cave molly females weigh less for a given standard length, have less body fat, invest more into reproduction, and produce larger but fewer offspring than surface females^29^, while mature cave molly males are larger, have less body fat, and invest less into reproduction, but weigh less for a given size than surface molly males^30^. However, whether or not these life history changes constitute local adaptation has so far not been examined in depth.

Here, we present an experiment in which we quantify the combined effects of development under high vs. low food availability and continuous darkness vs. normal light:dark photoperiod on the life histories of mollies derived from CdA and surrounding nontoxic surface streams. Specifically, we aim at identifying the degree to which cave mollies are locally adapted to a low-food and permanently dark cave environment. Thus, to fulfil the requirements for local adaptation with regards to ‘home versus away’ and ‘native versus foreign’^1^, we predicted cave mollies to have higher fitness than surface mollies when reared in continuous darkness and on low food rations—the experimental conditions best resembling their native habitat, CdA (‘home’)—but to have lower relative fitness when reared under either a different photoperiod or a different food regime (‘away’). Furthermore, we predicted the opposite trend for surface mollies, whose ‘home’ habitat should be characterized by high food availability coupled with a normal light:dark photoperiod.

## Results

### Ability to reproduce

**Figure 1:**
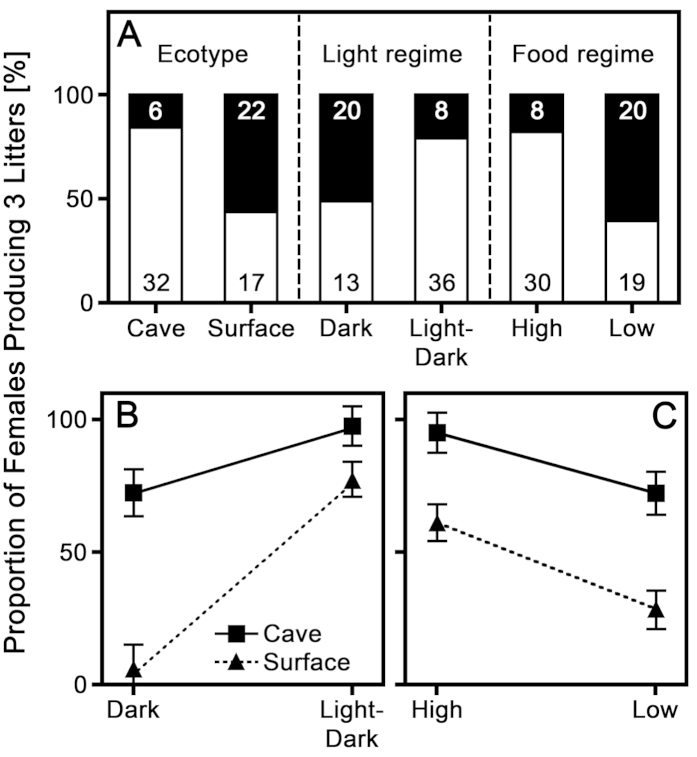
Visualization of the GLMM results on the proportion of female *Poecilia mexicana* (*N* = 77) that reached their full reproductive potential (i.e., gave birth to three litters) within the limits of our experiment. (**A**) The three significant main effects (ecotype, light regime and food regime) combined for cave and surface molly females. White represents the proportion of females that reached their full reproductive potential, black the proportion that failed to do so and numbers within the bars represent sample sizes. (**B**) Visualization of the significant interaction effect ‘ecotype-by-light regime’ and (**C**) the non-significant interaction effect ‘ecotype-by-food regime’ using estimated marginal means ± SEM.

All individuals (*N* = 64) that began male metamorphosis also successfully matured. This resulted in no measurable variation in the ability of males to attain maturity in the different experimental treatments, so we excluded them from our planned analysis. When analysing female reproduction (*N* = 77), the final model included significant contributions from ecotype (*F*_1,3_ = 171.962, *P* = 0.001), light regime (*F*_1,3_ = 181.674, *P* = 0.001) and food regime (*F*_1,3_ = 110.598, *P* = 0.002), as well as the interaction ecotype-by-light regime (*F*_1,3_ = 12.415, *P* = 0.039). Cave molly females were more successful in producing three consecutive litters than surface molly females (Fig. 1A). Moreover, there was a significant interaction between light regime and ecotype because permanent darkness had a much stronger negative effect on surface than cave mollies (Fig. 1B): In permanent darkness, 11 out of 17 cave molly females successfully gave birth to three consecutive broods, while only 1 out of 16 surface molly females successfully reproduced. In contrast, 20 out of 21 cave molly females and 16 of 23 surface molly females completed their life cycle in the 12:12 hr light:dark cycle (Supplementary Table S2).

When restricting this analysis to only those females of both ecotypes that were raised in the light:dark regime (*N* = 44), the final model consisted only of the factors ecotype and food regime, but neither attained statistical significance (P > 0.095 in both cases). More cave molly females successfully completed their life cycle than surface molly females (see above), but reproductive success was lower in the low food treatment (high food: 20 out of 21, low food: 16 out of 23; Supplementary Table S2).

### Multivariate analyses

In the mixed-model MANOVA on male life histories [i.e., standard length (SL), age at maturity, lean weight, fat content, gonadosomatic index (GSI), maturation time, and pre-maturation growth rate], all main effects (ecotype, light regime, and food regime) as well as the interaction of ecotype-by-food regime had significant effects (Table 1A). This significant interaction was mainly driven by ecotype-specific responses to food availability in age at maturity and maturation time (see univariate results below). For females [i.e., SL at 1^st^ parturition, SL at 3^rd^ parturition, age at 1^st^ parturition, lean weight, fat content, reproductive allocation (RA), pre-maturation growth rate, neonate SL, neonate dry weight, neonate fat content], all main effects had significant effects on life histories (Table 1B), but there were no significant interactions.

On the basis of these results, we proceeded to univariate analyses (Tables 2, 3, 4), and in the subsequent sections will then specifically evaluate patterns driven by individual factors within the univariate models.

### Univariate analyses: Differences between ecotypes

Male cave mollies were significantly smaller at maturity than surface mollies (estimated marginal means ± SEM for SL, cave: 20.68 ± 0.38 mm, surface: 21.46 ± 0.62 mm; lean weight at SL = 21.73 mm, cave: 0.035 ± 0.004 g, surface: 0.045 ± 0.003 g) and had a lower pre-maturation growth rate (cave: 0.062 ± 0.009 mm/day, surface: 0.070 ± 0.006 mm/day; Table 2; Supplementary Table S1). Cave mollies were also much older at maturity (cave: 164.99 ± 9.73 days, surface: 95.43 ± 5.74 days). For none of the ecotype-by-treatment combinations did the distribution of male SL at maturity differ significantly from normality (Shapiro-Wilk test: *P* > 0.146 in all cases).

For females we had to restrict our univariate comparison of life history traits between ecotypes to just those fish reared in the light:dark treatment because only one fish from the surface population reproduced in the dark treatment (Table 3; Supplementary Table S2). Cave and surface mollies did not differ significantly in age at 1^st^ parturition, but cave mollies were significantly smaller at 1^st^ parturition (cave: 28.55 ± 0.56 mm, surface: 30.18 ± 0.65 mm) and had slower pre-maturation growth rates (cave: 0.057 ± 0.003 mm/day, surface: 0.072 ± 0.003 mm/day). Ecotypes did not differ in the relative amounts of stored body fat, but cave mollies weighed less than surface mollies after the production of the third litter (dry weight at SL 33.44 mm, cave: 0.143 ± 0.004 g, surface: 0.220 ± 0.006 g). Their neonates were longer (cave: 10.41 ± 0.09 mm, surface: 8.62 ± 0.10 mm) and almost twice as heavy as surface molly neonates (at SL = 33.44 mm, cave: 6.620 ± 0.193 mg, surface: 3.808 ± 0.217 mg). Moreover, cave molly females invested proportionally more into the third litter than surface molly females (reproductive allocation, RA, cave: 17.68 ± 1.31%; surface: 11.70 ± 1.51%). However, the total dry mass of the third litter was slightly, albeit not significantly, larger in surface mollies (cave: 27.028 ± 4.549 mg, surface: 29.172 ± 3.144 mg; Mann-Whitney U-test: *N* = 36, *U* = 123.0, *P* = 0.35), so that the difference in RA was mainly driven by the observed differences in female dry weight rather than in an absolute increase in litter mass in cave mollies.

### Univariate analyses: The effects of light treatment

For males, permanent darkness led to maturation at a smaller size (dark: 20.03 ± 0.78 mm, light:dark: 22.11 ± 0.47 mm), less stored body fat (dark: 5.83 ± 1.54%, light:dark: 8.85 ± 1.17%), and slower growth (dark: 0.054 ± 0.009 mm/day, light:dark: 0.078 ± 0.006 mm/day) compared to males raised in the light room (Table 2).

For females, the strongest effect of permanent darkness was found for surface mollies raised in permanent darkness, because only one ever reproduced (see section on ‘ability to reproduce’ above). Compared to cave molly females raised in light, cave molly females raised in permanent darkness matured at an older age (age at 1^st^ parturition, dark: 299.42 ± 15.98 days, light: 255.13 ± 12.38 days), had slower pre-maturation growth rates (dark: 0.047 ± 0.004 mm/day, light: 0.057 ± 0.003 mm/day), were shorter at 3^rd^ parturition (estimated marginal means for SL at 3^rd^ parturition, dark: 29.99 ± 0.96 mm, light: 32.72 ± 0.76 mm), but weighed more for a given standard length (lean weight at SL = 31.97 mm, dark: 0.130 ± 0.011 g, light: 0.112 ± 0.008 g). They also had less body fat (dark: 1.90 ± 1.13%, light: 4.25 ± 0.92%), produced slightly shorter neonates (neonate SL at mother SL = 31.97 mm, dark: 10.01 ± 0.16 mm, light: 10.34 ± 0.10 mm), and had a smaller relative investment into their third litter (RA, dark: 10.93 ± 2.17%, light: 17.61 ± 1.53%; Table 4).

### Univariate analyses: The effects of resource availability (food regime)

Low food availability caused males to be shorter at maturity (high: 21.85 ± 0.50 mm, low: 20.30 ± 0.48 mm), have less body fat (high: 10.56 ± 1.17%, low: 4.12 ± 1.13%), grow more slowly prior to the onset of maturation (high: 0.090 ± 0.007 mm/day, low: 0.043 ± 0.007 mm/day), and take longer to mature (high: 35.44 ± 1.44 days, low: 39.16 ± 1.47 days), thus being older at maturity than males from the high-food regime (high: 114.29 ± 7.62 days, low: 146.13 ± 7.50 days; Table 2).

When females of both ecotypes from only the light:dark treatment were considered, the amount of available resources had a significant influence on age and SL at 1^st^ parturition, SL at 3^rd^ parturition, and the amount of stored fat reserves (Table 3). Compared to females from the low-food regime, females from the high-food regime were younger at 1^st^ parturition (high: 231.55 ± 9.66 days, low: 283.27 ± 10.87 days), larger at both 1^st^ and 3^rd^ parturition (1^st^ parturition, high: 31.35 ± 0.56 mm, low: 27.38 ± 0.65 mm; 3^rd^ parturition, high: 35.73 ± 0.67 mm, low: 30.47 ± 0.76 mm), had faster pre-maturation growth rates (high: 0.085 ± 0.003 mm/day, low: 0.044 ± 0.003 mm/day), and stored close to twice the amount of body fat (high: 5.64 ± 0.62%, low: 3.31 ± 0.69%).

When we instead analyse the data for only cave molly females in light:dark and constant darkness, cave molly females raised in the high-food regime were able to mature at a younger age (age at 1^st^ parturition, high: 242.97 ± 11.15 days, low: 311.58 ± 15.74 days), grew faster prior to maturation (high: 0.070 ± 0.003 mm/day, low: 0.034 ± 0.004 mm/day), and were of larger body size (SL at 1^st^ parturition, high: 29.92 ± 0.73 mm, low: 26.49 ± 1.03 mm). They were longer at 3^rd^ parturition than their counterparts from the low-food regime (SL at 3^rd^ parturition, high: 33.69 ± 0.69 mm, low: 29.02 ± 0.94 mm) and were able to store more body fat (fat content at 3^rd^ parturition, high: 4.95 ± 0.84%, low: 1.20 ± 1.11%; Table 4).

### Univariate analyses: Interaction effects

**Figure 2:**
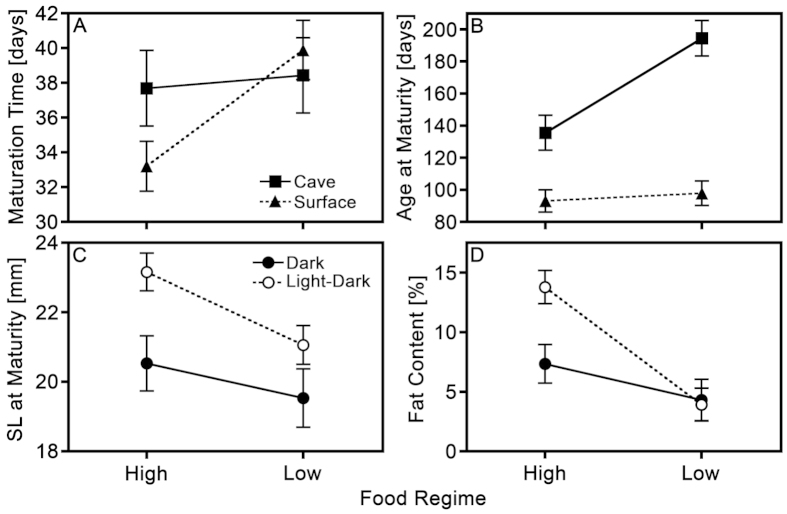
Multipanel graphical representation of the interaction effects from the mixed-model ANOVAs on male life histories. (**A,B**) are visualizations of the significant interaction effects of ‘ecotype-by-food regime’ on maturation time and age at maturity, respectively, while (**C**,**D**) are visualizations of the non-significant trends for interaction effects of ‘light regime-by-food regime’ on SL at maturity and fat content. Depicted are estimated marginal means ± SEM.

Univariate mixed models uncovered significant interaction effects of ‘ecotype-by-food regime’ on maturation time and age at maturity in males (Table 2), because the effect of food regime on maturation time was stronger in surface mollies but the effect of food regime on age at maturity was stronger in cave mollies (Fig. 2A,B). The interaction effects of light regime-by-food regime on SL and fat content at maturity, however, were only suggestive (i.e., 0.05 < *P* < 0.1; Table 2; Fig. 2C,D), while all other interactions were not significant.

**Figure 3:**
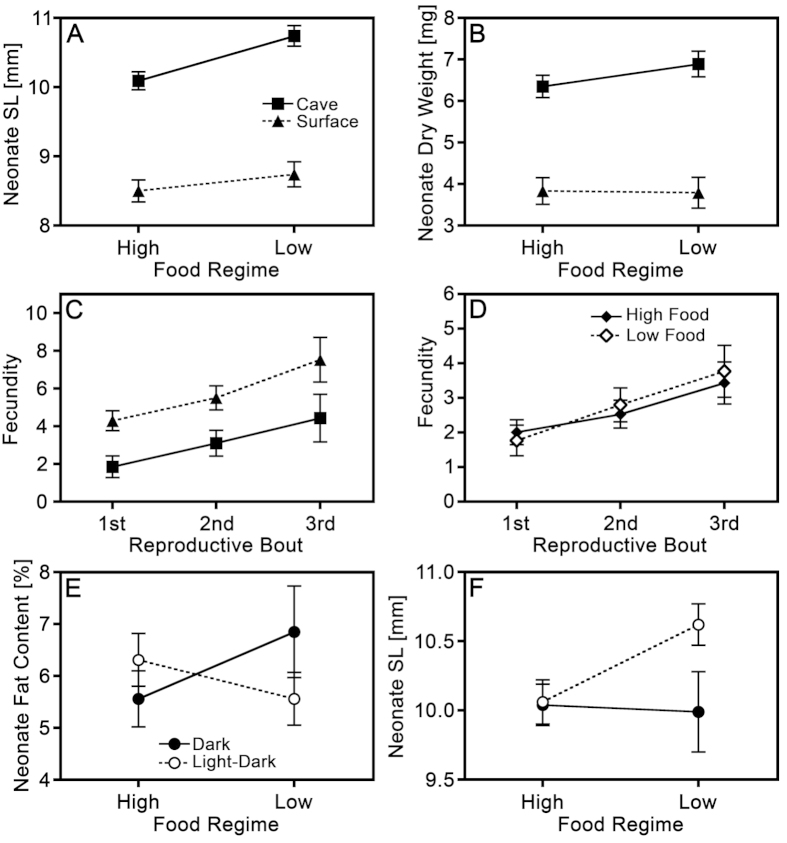
Multipanel graphical representation of the interaction effects from the mixed-model ANOVAs (**A,B,E,F**) and mixed-model rmANOVAs (**C,D**) on female life histories. (**A–C**) are visualizations of the analyses of cave and surface molly females raised in the light:dark regime only, while (**D–F**) are visualizations of the analyses of cave molly females raised in both the dark and the light:dark regime. Depicted are estimated marginal means ± SEM.

For cave and surface females raised under a light:dark cycle, there was a significant interaction of food availability-by-ecotype for offspring length: cave mollies increased offspring length more in response to low-food availability than did surface mollies (Fig. 3A). A similar, albeit not significant, tendency was uncovered for neonate dry weight (Table 3; Fig. 3B). Both of these patterns suggest that cave molly females are locally adapted to a low-food environment.

In the analysis of cave mollies from both light regimes, there were also two non-significant trends suggesting an interaction of light regime-by-food availability. Cave molly females in the darkroom decreased neonate SL in the low-food regime coupled with an increase in neonate fat content, while both patterns were reversed in the light regime (Table 4; Fig. 3E,F).

### Repeated measures analyses: Reproductive bout-specific changes in fecundity and interbrood interval

Females from both populations produced more offspring in each successive litter. Fecundity was higher in surface females but also increased more rapidly in successive litters, causing a significant ‘reproductive bout-by-ecotype’ interaction (Fig. 3C,D). There were no significant effects of any factor on interbrood intervals (see Supplementary Results for details). Our analysis of cave molly females under light:dark versus dark conditions at high and low food availability revealed that only the interaction of ‘reproductive bout-by-food regime’ had a significant effect on fecundity. Interbrood intervals of cave mollies were also significantly longer in the light:dark treatment compared to those of cave mollies from the darkroom. (see Supplementary Results for details).

## Discussion

Life histories of lab-born cave molly offspring reared under controlled laboratory conditions largely conformed to those reported for other cave animals^13^,^31^,^32^. Reduced growth rates and the lower size-specific lean weight at maturity in both sexes suggest heritable differences in physiology, and indeed, a recent study found cave mollies to have higher resting metabolic rates than similar-sized surface mollies^33^. This may contribute to cave mollies being less efficient in converting food into biomass than surface mollies when both received the same food rations. However, we did not find consistent differences in body fat or interbrood intervals between both ecotypes, which could indicate that the presence of H_2_S in CdA is the main driver for the low body fat characteristic for wild-caught cave mollies^29^,^30^.

Heritability of population differences should be interpreted with caution as we used first generation laboratory-reared offspring so that some of these observed patterns could be influenced by maternal^34^,^35^ as well as epigenetic effects^36^. However, several lines of evidence support heritable differences between cave and surface mollies. First, previous studies using > 3^rd^ generation laboratory-reared *P. mexicana* from our greenhouse and laboratory stock tanks revealed similar results (fecundity^37^; offspring size^38^; male lean weight^30^). Second, population differences in most life-history traits identified for cave and surface mollies have been demonstrated to be heritable in other poeciliid systems (e.g., *Poecilia reticulata*^39^; *Brachyrhaphis rhabdophora*^40^; *Gambusia hubbsi*^41^).

For cave mollies, permanent darkness and low food availability most closely resemble the ‘local’ conditions experienced in CdA, while higher food availability and our experimental 12:12 hr light:dark cycle represent a ‘foreign’ environment; for surface mollies, the pattern is opposite. We uncovered sex-specific responses to ‘local versus foreign’ experimental conditions, because surface molly females, but not males, suffered from almost complete reproductive failure when raised in darkness (i.e., the foreign environment), irrespective of food treatment. Previously, we demonstrated that this effect was for the most part due to surface molly females contracting the fatal, and stress-related columnaris disease in darkness^42^, which led to subsequent mortality in more than 80% of detected cases in this common garden experiment^43^.

We also uncovered two patterns that at first sight appear opposite to expectation under the ‘local versus foreign’ paradigm. First, low food availability caused a much larger increase in age at maturity for cave molly males compared to surface molly males, which is surprising if we assume individual fitness to increase as the age at maturity declines. Unfortunately, we have no way of quantifying the relative impact of age at maturity on male fitness in this system, so we cannot gauge from these results whether they are indeed in conflict with the ‘local versus foreign’ paradigm. It is tempting to speculate though that the effects of size at maturity might outweigh the effects of age at maturity, which, if true, would conform to the ‘local versus foreign’ paradigm for low food availability in males of both ecotypes.

Second, a larger proportion of cave than surface molly females successfully reproduced three times under light:dark conditions. While this seems to indicate a pattern opposite to the one expected under the ‘local versus foreign’ paradigm, our results are actually more complex: Most surface molly females that failed to successfully reproduce three times in the light:dark treatment were raised under low-food availability (see Supplementary Table S3; despite a non-significant ecotype-by-food interaction), a condition we predicted cave but not surface mollies to be locally adapted to. Therefore, it appears that with regards to successful third reproduction, cave mollies indeed behave according to ‘local’ conditions also in the low-food/light:dark treatment. This interpretation is further supported by the fact that, while all mollies responded to low food rations by producing larger offspring (measured as SL at birth), the magnitude of this response was significantly larger in cave mollies—a response known to be adaptive in low-resource environments (e.g., *P. reticulata*^44^,^45^; least killifish, *Heterandria formosa*^46^). However, it is important to note that surface molly females under all conditions always had a higher fecundity than cave molly females, effectively granting them a higher fitness in the high-food/light:dark treatment, which best resembles their native habitat (i.e., congruent with ‘local versus foreign’).

Patterns for ‘home versus away’, on the other hand, were much less clear-cut. For surface molly females, this framework clearly also applied, as they performed best in the high-food/light:dark treatment (i.e., the ‘home’ treatment), but suffered significant fitness reductions primarily through increased mortalities and reproductive failures in all other treatments, including the low-food/light:dark treatment. Cave molly females, on the other hand, actually had a higher fitness in the ‘away’ treatments relative to the ‘home’ treatment. However, while we find the distinction between the two different components of local adaptation *sensu* Kawecki & Ebert^1^ (‘home vs. away’ and ‘local vs. foreign’) generally helpful, we would argue that (*a*) this can only be fully evaluated if all aspects of fitness can properly be assessed, and (*b*) this is a difficult conceptual framework to apply to organisms colonizing an extreme habitat. Regarding the first point, we concentrated here on correlates of fitness that can be assessed via life-history analyses, but our study design did not enable us to evaluate the interplay between divergent life histories and, for example, predation, competition, or even offspring survival under natural conditions. Moreover, one characteristic of an extreme habitat is that it should result in a net fitness loss of any organism entering it^47^,^48^. On this premise, any reduction of this imposed fitness cost should be an indication of local adaptation to this extreme environment, even if overall fitness might still be higher in an environment in which the extreme conditions are lacking. Reciprocal transplant experiments—measuring 24 h mortality as a correlate of individual fitness—of surface- and cave-dwelling *P. mexicana* adapting to toxic H_2_S support this view: while fish from non-sulphidic habitats experienced high mortality when transferred into sulphidic (‘away’) habitats, mortality in the opposite direction was low in sulphide-adapted fish from two out of three drainages^19^,^49^.

How can this strong, sex-specific pattern of differences between ecotypes in reproduction and survival be explained? With regards to the response to permanent darkness, surface mollies, as visually-oriented organisms, might not have been able to forage effectively in permanent darkness and were thus starved^50^,^51^. However, we find this explanation unlikely, as we did not uncover a significant interaction of ecotype-by-light regime on male growth rate, fat content, or lean weight that would have indicated that male surface mollies had difficulties in acquiring resources in darkness; in contrast, surface molly males actually sustained higher growth rates than cave mollies receiving the same treatment. Moreover, even for those females that failed to reproduce, growth was normal until termination of the experiment or until contraction of columnaris disease (Supplementary Table S3).

We propose a different hypothesis to explain this pattern: Exposure to light in general, and differences in photoperiod in particular, are known to be important for the regulation of melatonin secretion and affect oocyte growth and maturation in teleost fishes^52^. Surface molly females raised in permanent darkness likely lacked the appropriate photoperiod/exposure-to-light cue necessary to trigger successful reproduction. This would also explain why only female, but not male surface mollies failed to reproduce in permanent darkness. Similarly, with regards to the proportion of body weight that constitutes reproductive tissues (i.e., testes in males versus ovaries, oocytes, and embryos in females), males make a much smaller investment into reproduction than females. In natural populations, of course, this might be balanced out by other costs of reproduction (i.e., energetic costs related to searching for mates, courtship, sneaking, and intrasexual aggression)^53^. However, our experimental setup, in which fish were raised in isolation and males were removed once they reached sexual maturity, largely precluded such costs from arising. Therefore, it is likely that the reduced food availability in the low-food regimen led to higher rates of stress in non-adapted surface females than in surface males, ultimately resulting in the higher proportion of reproductive failures and higher mortality observed in surface molly females even under light:dark conditions.

Finally, we were able to demonstrate that the typical life histories described for cave animals (e.g., reduced growth rates and delayed maturation) are not solely an adaptation to low resource availability as previously assumed^31^,^32^, but rather could be driven by the combination of low resource availability coupled with permanent darkness. Our study further demonstrates that ‘cave phenotypes’ can be the result of both heritable population differences and plastic responses to different ecological conditions. However, life-history responses to darkness might not necessarily be adaptations but could simply be constraints imposed by the absence of light interfering with physiological pathways, as uncovered for female reproduction. It is important to keep in mind that *P. mexicana* are primarily visually-oriented fish, and so the strong influence of permanent darkness in shaping life histories uncovered here might be weaker in nocturnal, or low-light adapted species like catfishes (Siluriformes) who comprise approximately 30% of all cave fishes^13^,^50^. Additional life-history studies on representatives of those taxa will have to investigate this further. On a larger scale, our data provide the first experimental evidence for the strong selection by permanent darkness and low-food availability on a visually-oriented surface fish, and help explain why most cave-adapted species are usually derived from either nocturnal organisms or organisms already pre-adapted to a low-light environment as experienced, for example, in highly turbid waters^43^,^50^,^51^.

In conclusion, we uncovered strong evidence for sex-specific local life-history adaptation in both surface- and cave-dwelling *P. mexicana*. This suggests that migrant females between the cave and surface habitats in natural populations will suffer from decreased fitness in the ‘foreign’ habitat compared to the performance of locally adapted ‘native’ females, which represents a significant barrier to gene exchange between the two populations. While males are not affected to the same extent, previous studies suggest that they (i.e., surface molly males within the cave and cave molly males in surface habitats) will be at a strong disadvantage during mate choice in the non-native habitat^54^,^55^. Migrants of both sexes also suffer high mortalities due to the presence and absence of toxic H_2_S^19^ and migrant-specific predation^56^ between the cave and surface habitats in this system. Hence, this study supports our previous hypothesis^29^ that divergent life histories in this system act as an additional mechanism that, along with trophic^57^, morphological^18^, and behavioural divergence^25^,^58^, as well as divergent toxicity^19^- and predator regimes^56^, effectively restricts gene flow through direct selection against ‘migrants’^16^,^17^. In other words, disruptive life-history trait evolution due to local adaptations to different habitat types provides another mechanistic link promoting ecological diversification and, ultimately, parapatric (ecological) speciation in this, and likely also in other cave systems^13^. This is strong evidence against the argument that niche conservatism and local adaptation could be preventing the initial stages of speciation by facilitating gene flow^59^. On the contrary, our study provides further support to the notion that ecological speciation will be facilitated by local adaptation even in the absence of physical barriers, as long as divergent selection between the two interconnected habitats is of sufficient strength^60^. This is exemplified by the numerous examples of ecological speciation facilitated by strong divergent selection as a result of the colonization of various extreme habitats (e.g., in toxic^23^ or low-oxygen environments)^61^, of which caves are but one example.

## Material and Methods

### Common-garden protocol

For the collection of these data, the authors have adhered to the Guidelines for the Use of Animals in Research. The experiment reported here was performed in accordance with the respective laws in the USA and Mexico. Specifically, all necessary permits for the collection of live specimens from natural populations in Mexico were obtained (Permiso de Pesca de Fomento: DGOPA.06192.240608.-1562), and the experimental protocols were approved by the University of Oklahoma Institutional Animal Care and Use Committee (AUS-IACUC: R06-026).

Experimental subjects were first generation laboratory-born fish derived from field-caught individuals collected in the Río Tacotalpa drainage in Tabasco, southern México. Sexually mature surface- and cave-dwelling *P. mexicana* males and females were collected in January 2009 from chamber V of CdA^14^ as well as from two surface habitats of the same drainage (Arroyo Bonita and Río Amatan)^22^. These field-caught fish were transported to the University of Oklahoma and were housed in several mixed-sex tanks, in which they were exposed to identical environmental conditions (natural light:dark cycle, and no hydrogen sulphide or predators present). Pregnant females showing a distended abdomen were isolated in individual 10-L aquaria, fed *ad libitum* amounts of commercially available flake food, and checked twice daily for offspring until they had given birth. Only one brood per female was included in the actual experiment.

Females were removed from their tanks on the day of birth and measured for standard length (SL) to the nearest millimetre. Fry were raised together at a maximum density of five offspring per 10-L tank for 37 days under *ad-libitum* food- (brine shrimp and ground-up flake food) and benign (non-sulphidic) water conditions; partial water changes were performed every second day. Large broods (≥10 offspring) were separated into two groups of five offspring per tank. In total, we thus raised *N* = 44 broods [22 from surface (11 from AB and 11 RA) and 22 from cave mollies].

After 37 days, we randomly selected up to four offspring from each brood and randomly assigned them to one of four treatments. However, cave mollies regularly give birth to less than four offspring^29^, so in this case we selected all offspring available from that particular brood up to a maximum of four (again, these offspring were randomly assigned to the four different treatments but for the last couple of broods randomness was sometimes constrained to ensure roughly equal numbers in each treatment group), leading to an overall sample size of 145 individuals in the experiment. These 145 individuals were then raised to sexual maturity (in case of males) or until they had given birth to their third brood (in case of females). We measured standard length and weight of each individual every two weeks on the day we performed a water change. Fish were kept in their respective treatments until they were (*a*) sexually mature (males), (*b*) had produced three consecutive broods of young (females), or (*c*) 1 year of age without successful reproduction, at which point they were classified as having failed to reproduce.

Generally, our common garden experiment followed well-established protocols^62^,^63^, but some changes were made to pursue specific questions in the cave molly complex: *Treatments 1* and *2* involved a 12:12 h light:dark cycle coupled with low (*tr. 1*) or high food availability (*tr. 2*). In *treatments 3* and *4*, fish were raised in perpetual darkness, yet again under low (*tr. 3*) or high food (*tr. 4*). Placement of each fish within the laboratory setup was also random, but fish from the same brood were never placed upon the same shelf (nonetheless, shelf identity was included as the random variable ‘block’ in all statistical analyses). Feeding regimes also followed established protocols^62^,^63^, but were adjusted to fit mollies according to experience during trial runs (R. Riesch, unpubl. data). For example, the original protocols published by Reznick^62^ and Reznick & Yang^63^ were based on feeding measured amounts of liver paste, yet our preliminary studies showed that mollies would not grow well on liver paste (R. Riesch, unpubl. data), so we exchanged liver paste for *Daphnia*. Hence, fish were fed twice daily with a Hamilton micropipette: measured amounts of newly hatched *Artemia* nauplii in the morning and *Daphnia* in the evening. Food levels were increased every two weeks.

No method has been established to visually determine the sex of immature mollies. Upon entering the maturation process, males undergo morphogenetic changes as their anal fin transforms into an intromittent organ, the gonopodium^64^,^65^,^66^. Even though there are slight differences among species, the general metamorphosis is similar to that described by Turner^64^ for *Gambusia affinis* and Greven^65^ for *Poecilia reticulata*. To define the endpoint of anal fin metamorphosis for *P. mexicana*, we also consulted the illustrations of the fully developed gonopodium of several *Poecilia* spp. presented by Rosen and Bailey^67^ as well as photographs of mature *P. mexicana* males from a previous study^30^. Hence, for males, the experiment ended when anal fin metamorphosis was complete (i.e., the gonopodium became largely translucent, the distal tip was pointed, and the distal hook had fully developed; see Supplementary Figure S1). Males were sacrificed with an overdose of anaesthetic (MS-222) and preserved in 10% formalin on the day they reached sexual maturity. In the case of females, there are no obvious outward signs of sexual maturity, so putative females were mated once a week with a mature male of their population from our stock tanks as soon as they reached a size of 24 mm, as previous field studies have shown that the minimum size of reproducing wild-caught *P. mexicana* females is around 30 mm^29^. Females were therefore only scored as ‘reproductively active’, if they successfully produced a brood of offspring within their first year of life. We measured length and mass of females after each litter, then sacrificed and preserved the females immediately after they produced their third litter. All offspring from litters 1 through 3 were also sacrificed and preserved immediately after birth.

Males and females from the experiment were dissected as described in Reznick and Endler^68^ and Riesch *et al.*^29^,^30^. In short, reproductive tissues, which often included yolking ova for the next litter in females, were separated from somatic tissues. Somatic and reproductive tissues (for dissected adults), as well as all preserved offspring from broods 1–3 were then dried for 24 hours at 55 °C and reweighed. To assess adult and offspring condition, somatic tissues (and, if present, any developing embryos from the dissected females) were rinsed six times for at least six hours in petroleum ether to extract soluble non-structural fats^69^,^70^, then redried and reweighed. We calculated reproductive allocation (RA) for females by dividing offspring dry weight by the sum of offspring dry weight plus somatic dry weight^66^, and gonadosomatic index (GSI) for males by dividing testis dry weight by the sum of testis dry weight plus somatic dry weight^30^.

We thus collected data on the following female life-history traits: age at 1^st^ parturition [days], standard length (SL) at 1^st^ and 3^rd^ parturition [mm], as well as dry and lean weight [g], fat content [%], and RA [%] at 3^rd^ parturition. Furthermore, we measured fecundity [number of offspring], neonate standard length [mm], neonate dry and lean weight [mg], and neonate fat content [%] for broods 1–3, quantified interbrood intervals [days] (between the 1^st^ and 2^nd^ as well as between the 2^nd^ and 3^rd^ brood), and calculated growth rates [mm/d] prior to the onset of reproduction. Growth rates [mm/d] were based on length measurements taken ≥ 31 days prior to first parturition; this cut-off was chosen *a posteriori* based on average interbrood intervals.

For males, we collected data on SL at maturity [mm], age at maturity [days], dry and lean weight at maturity [g], fat content at maturity [%], GSI at maturity [%], maturation time [days] (i.e., the time it took from the first indication of anal fin metamorphosis to the fully developed gonopodium), and pre-maturation growth rate (only including length measurements taken prior to the onset of anal fin metamorphosis).

We log_10_-transformed all length, weight, and time measurements, arcsine(square root)-transformed all percentages, square root-transformed fecundity, and then subsequently *z*-transformed all variables to meet assumptions of statistical analyses (i.e., these transformations greatly facilitated normality of model residuals). To remove size/allometry effects on life-history traits other than SL, we screened these variables for covariance with SL by regressing them against SL separately for each sex, confirmed homogeneity of slopes among ecotypes (*P* > 0.35 in all cases), and in case of significant regressions used residuals from these models in all subsequent analyses (this applied to female and male lean weight, fecundity, average neonate SL, average neonate dry weight, and average interbrood interval).

### Statistical Analyses: Ability to reproduce

To analyse differences in fitness of potential migrants between different light and food regimes, we compared ‘full reproductive potential’ (i.e., sexual maturity in males and three successful litters in females) by using a Generalized Linear Mixed Model (GLMM) with a binominal error distribution and a logit-link function. ‘Ability to reproduce’ (binary data: 1 = achieved; 0 = not achieved) was used as the dependent variable, and we included ‘ecotype (cave vs. surface)’, ‘food regime’, as well as ‘light regime’ as fixed factors. However, including ‘mother ID’ (to control for differences between pedigrees) and ‘block nested within room’ [hereafter ‘block(room)’; i.e., what shelf a tank was on in each room] as random factors (either alone or in combination) always prevented the final Hessian matrix from being positive definite, so we did not include these random factors in this analysis. All possible second order interactions of the fixed factors were included in the initial model, but non-significant interaction terms were removed in a stepwise elimination procedure (*P* > 0.7 in all cases). This analysis was conducted in IBM SPSS Statistics for Mac, Version 20.0.0 (IBM Corp., Armonk, NY). Two surface molly females and one cave molly female were scored as not having achieved their full reproductive potential for this analysis, despite their having produced at least one litter prior to the end of the experiment after 18 months. The cave molly female contracted a severe eye infection; we euthanized the female before this infection resulted in premature death. The two surface mollies had abnormally long intervals between their first litter and the end of the experiment (158 days and 76 days, respectively) suggesting that they had either ceased reproducing or had an abnormally long interbrood interval (see suppl. Tables S2 and S3). Mortality rates and occurrence of the stress-related columnaris disease^42^ in this same experiment were described in a previous publication^43^, while we focus on new variables (1^st^ through 3^rd^ reproduction) here. Moreover, post-hoc dissections revealed that all females that failed to reproduce, and for which advanced stages of columnaris disease had not rendered the internal organs unidentifiable, had well-developed ovaries but simply lacked yolking oocytes or developing embryos (R. Riesch, unpublished data).

### Statistical Analyses: Multivariate and univariate models

Our primary test for differential responses of the two *P. mexicana* ecotypes to the experimental treatments, and ultimately local adaptation, was a mixed-model multivariate analysis of variance. Sexes were analysed separately. Phenotypic traits served as dependent variables; all traits described above were included for males, while for females we included age and SL at 1^st^ parturition, pre-maturation growth rate, as well as SL, lean weight, fat content, and RA at 3^rd^ parturition. Fecundity, neonate SL, neonate dry weight, neonate fat content, and interbood interval were averaged across the three litters. We tested for effects of ‘ecotype’, ‘light regime’, and ‘food regime’ while including ‘mother ID’ and ‘block(room)’ as random effects. Statistical significance for the main effects and any main-effect interactions (all possible interactions were tested for males, but for females we only included the interaction of ‘ecotype-by-food regime’, because only one surface molly female successfully reproduced in darkness) was determined with *F-*tests using restricted maximum likelihood and the Kenward–Roger degrees of freedom adjustment^71^ to appropriately test the fixed effects while treating ‘mother ID’ and ‘block(room)’ as random terms. This significance test was conducted using the MIXED procedure in SAS v 9.3 (SAS Institute, Cary, NC, USA; for a sample code see^41^).

Once multivariate significance was detected, we ran post-hoc mixed-model univariate analyses of variance separately for each male and female life-history trait to identify how differences between ecotypes and experimental treatments specifically affected each trait. All univariate models were run using R (2.15.1) and were conducted by means of a general linear model (GLM) fit using the R package lme4^72^ that fits random effects using restricted maximum likelihood. The models for male life-history traits were similar in structure to the multivariate model described above. The models for female life histories, however, differed slightly from the multivariate model because we excluded the only surface molly female that successfully reproduced when raised in permanent darkness (see results section). Also some life-history traits (i.e., fecundity and interbrood interval) were more appropriately analysed in a repeated measures design (see below). Thus, we ran two separate sets of univariate mixed-models for female life-history traits (i.e., age and SL at 1^st^ parturition, pre-maturation growth rate, SL, lean weight, fat content, and RA at 3^rd^ parturition, average neonate SL, dry weight, and fat content): (1) The first set of models was restricted to females raised under a light:dark cycle and included the factors ‘ecotype’, ‘food regime’, and the interaction of ’ecotype-by-food regime’, while (2) the second set was restricted to cave molly females and included the factors ‘light regime’, ‘food regime’, and the interaction of ‘light regime-by-food regime’. Both sets of models further included ‘mother ID’ and ‘block’(for the analysis of fish raised in light) or ‘block(room)’ (for the cave molly-only analysis) as random effects. For significant model terms we present estimated marginal means that were derived from simplified (i.e., no random effects) but otherwise similar models run in IBM SPSS Statistics for Mac, v 20.0.0 (IBM Corp., Armonk, NY).

Finally, we used univariate mixed-model repeated measures ANOVAs to investigate differences in fecundity (three levels, 1^st^ vs. 2^nd^ vs. 3^rd^ parturition) and interbrood interval (two levels, 1^st^ vs. 2^nd^ interbrood interval) between ecotypes and experimental treatments (see Supplementary Material and Methods for details).

Ethics Statement. This study was conducted under the University of Oklahoma Institutional Animal Care and Use Committee (IACUC #R06-026).

## Additional Information

**How to cite this article**: Riesch, R. *et al.* Sex-specific local life-history adaptation in surface- and cave-dwelling Atlantic mollies (*Poecilia mexicana*). *Sci. Rep.* **6**, 22968; doi: 10.1038/srep22968 (2016).

## Supplementary Material

Supplementary Information

## Figures and Tables

**Table 1:** Results from the mixed-model nested MANOVAs on differentiation of life-history traits of two *Poecilia mexicana* ecotypes.

Factor	*F*	*df*	*P*
(A) Males			
Ecotype	32.88	9, 222	<0.0001
Light regime	5.01	9, 222	<0.0001
Food regime	17.70	9, 222	<0.0001
Ecotype × food regime	2.80	9, 222	0.004
(B) Females			
Ecotype	16.86	5, 142	<0.0001
Light regime	2.92	5, 142	0.015
Food regime	19.39	5, 142	<0.0001

Fixed effects were ‘ecotype (cave vs. surface)’, ‘light regime (dark vs. light:dark)’, and ‘food regime (high vs. low)’, while ‘mother ID’ and ‘block(room)’ were included as random effects. (A) Male-only analysis, and (B) female-only analysis.

**Table 2:** Results from the mixed-model nested ANOVAs on differentiation of life-history traits of male *Poecilia mexicana*.

Trait	Ecotype	Light regime	Food regime
Growth rate [mm/d]	***χ*^2^ = 14.114, *P* = 0.0002**	***χ*^2^ = 3.939, *P* = 0.047**	***χ*^2^ = 6.981, *P* = 0.0082**
Maturation time [d]	*χ*^2^ = 1.451, *P* = 0.23	*χ*^2^ = 0.005, *P* = 0.95	***χ*^2^ = 5.907, *P* = 0.015**
Age at maturity [d]	***χ*^2^ = 149.991, *P* < 0.0001**	*χ*^2^ = 2.075, *P* = 0.15	***χ*^2^ = 19.475, *P* < 0.0001**
SL at maturity [mm]	***χ*^2^ = 7.782, *P* = 0.0053**	***χ*^2^ = 15.849, *P* < 0.0001**	***χ*^2^ = 18.741, *P* < 0.0001**
Lean weight at maturity [g]	***χ*^2^ = 4.483, *P* = 0.034**	*χ*^2^ = 0.944, *P* = 0.33	*χ*^2^ = 0.018, *P* = 0.89
Fat content at maturity [%]	*χ*^2^ = 2.389, *P* = 0.12	***χ*^2^ = 8.577, *P* = 0.0034**	***χ*^2^ = 34.922, *P* < 0.0001**
GSI at maturity [%]	*χ*^2^ = 1.153, *P* = 0.28	*χ*^2^ = 0.537, *P* = 0.46	*χ*^2^ = 0.290, *P* = 0.59
**Trait**	**Ecotype × Light regime**	**Ecotype × Food regime**	**Light regime × Food regime**
Growth rate [mm/d]	(*χ*^2^ = 0.358, *P* = 0.55)	(*χ*^2^ = 1.147, *P* = 0.28)	(*χ*^2^ = 0.115, *P* = 0.73)
Maturation time [d]	(*χ*^2^ = 0.013, *P* = 0.91)	***χ*^2^ = 4.196, *P* = 0.041**	(*χ*^2^ = 1.299, *P* = 0.25)
Age at maturity [d]	(*χ*^2^ = 1.165, *P* = 0.28)	***χ*^2^ = 11.468, *P* = 0.0007**	(*χ*^2^ = 0.095, *P* = 0.76)
SL at maturity [mm]	(*χ*^2^ = 1.507, *P* = 0.22)	(*χ*^2^ = 0.107, *P* = 0.74)	*χ*^2^ = 2.862, *P* = 0.091
Lean weight at maturity [g]	(*χ*^2^ = 0.394, *P* = 0.53)	(*χ*^2^ = 0.562, *P* = 0.45)	(*χ*^2^ = 0.622, *P* = 0.43)
Fat content at maturity [%]	(*χ*^2^ = 1.044, *P* = 0.31)	(*χ*^2^ = 0.734, *P* = 0.39)	*χ*^2^ = 2.846, *P* = 0.092
GSI at maturity [%]	(*χ*^2^ = 0.714, *P* = 0.40)	(*χ*^2^ = 0.028, *P* = 0.87)	(*χ*^2^ = 2.345, *P* = 0.13)

Fixed effects were ‘ecotype (cave vs. surface)’, ‘light regime (dark vs. light:dark)’ and ‘food regime (high vs. low)’, while ‘mother ID’ and ‘block(room)’ were included as random effects. Significant test statistics are in bold and test statistics in parentheses refer to non-significant interactions that were removed from the final model.

**Table 3:** Results from the mixed-model nested ANOVAs on differentiation of life-history traits of cave and surface molly females raised under a light:dark cycle.

Trait	Ecotype	Food regime	Ecotype × Food regime
Growth rate [mm/day]	***χ*^2^ = 12.326, *P* = 0.0005**	***χ*^2^ = 98.302, *P < *0.0001**	(*χ*^2^ = 1.801, *P* = 0.18)
Age at 1^st^ parturition [d]	*χ*^2^ = 0.895, *P* = 0.34	***χ*^2^ = 16.073, *P* < 0.0001**	(*χ*^2^ = 0.207, *P* = 0.65)
SL at 1^st^ parturition [mm]	***χ*^2^ = 4.648, *P* = 0.031**	***χ*^2^ = 19.608, *P* < 0.0001**	(*χ*^2^ = 1.477, *P* = 0.22)
SL at 3^rd^ parturition [mm]	*χ*^2^ = 1.794, *P* = 0.18	***χ*^2^ = 32.442, *P* < 0.0001**	(*χ*^2^ = 1.249, *P* = 0.26)
Lean weight at 3^rd^ parturition [g]	***χ*^2^ = 46.671, *P* < 0.0001**	*χ*^2^ = 0.606, *P* = 0.44	(*χ*^2^ = 1.801, *P* = 0.18)
Fat content at 3^rd^ parturition [%]	*χ*^2^ = 0.253, *P* = 0.62	***χ*^2^ = 10.261, *P* = 0.0014**	(*χ*^2^ = 0.304, *P* = 0.58)
RA at 3^rd^ parturition [%]	***χ*^2^ = 10.108, *P* = 0.0015**	*χ*^2^ = 0.0004, *P* = 0.98	(*χ*^2^ = 1.041, *P* = 0.31)
Neonate SL [mm]	***χ*^2^ = 85.548, *P* < 0.0001**	*χ*^2^ = 0.0002, *P* = 0.99	*χ*^2^ = 3.850, *P* = 0.050
Neonate dry weight [mg]	***χ*^2^ = 57.685, *P* < 0.0001**	*χ*^2^ = 2.046, *P* = 0.15	*χ*^2^ = 3.665, *P* = 0.055
Neonate fat content [%]	*χ*^2^ = 2.880, *P* = 0.090	*χ*^2^ = 0.985, *P* = 0.32	(*χ*^2^ = 0.002, *P* = 0.97)

Fixed effects were ‘ecotype (cave vs. surface)’ and ‘food regime (high vs. low)’, while ‘mother ID’ and ‘block(room)’ were included as random effects. Significant test statistics are in bold; test statistics in parentheses refer to non-significant interactions that were removed from the final model.

**Table 4:** Results from the mixed-model nested ANOVAs on differentiation of life-history traits of cave molly females raised under different light and food regimes.

Trait	Light regime	Food regime	Light regime × Food regime
Growth rate [mm/day]	***χ*^2^ = 5.244, *P* = 0.022**	***χ*^2^ = 62.365, *P* < 0.0001**	(*χ*^2^ = 0.027, *P* = 0.87)
Age at 1^st^ parturition [d]	***χ*^2^ = 4.737, *P* = 0.030**	***χ*^2^ = 17.584, *P* < 0.0001**	(*χ*^2^ = 0.127, *P* = 0.72)
SL at 1^st^ parturition [mm]	*χ*^2^ = 0.102, *P* = 0.75	***χ*^2^ = 11.238, *P* = 0.0008**	(*χ*^2^ = 0.002, *P* = 0.97)
SL at 3^rd^ parturition [mm]	***χ*^2^ = 6.658, *P* = 0.010**	***χ*^2^ = 27.378, *P* < 0.0001**	(*χ*^2^ = 1.094, *P* = 0.30)
Lean weight at 3^rd^ parturition [g]	***χ*^2^ = 4.278, *P* = 0.039**	*χ*^2^ = 2.400, *P* = 0.12	(*χ*^2^ = 0.027, *P* = 0.87)
Fat content at 3^rd^ parturition [%]	***χ*^2^ = 5.031, *P* = 0.025**	***χ*^2^ = 13.961, *P* = 0.0002**	(*χ*^2^ = 1.758, *P* = 0.18)
RA [%]	***χ*^2^ = 7.418, *P* = 0.0065**	*χ*^2^ = 1.514, *P* = 0.22	(*χ*^2^ = 0.194, *P* = 0.66)
Neonate SL [mm]	***χ*^2^ = 4.749, *P* = 0.029**	*χ*^2^ = 0.0041, *P* = 0.95	*χ*^2^ = 3.204, *P* = 0.074
Neonate dry weight [mg]	*χ*^2^ = 0.221, *P* = 0.64	*χ*^2^ = 0.148, *P* = 0.70	(*χ*^2^ = 0.088, *P* = 0.77)
Neonate fat content [%]	*χ*^2^ = 0.004, *P* = 0.95	*χ*^2^ = 0.073, *P* = 0.79	*χ*^2^ = 2.725, *P* = 0.099

Fixed effects were ‘light regime (dark vs. light:dark)’ and ‘food regime (high vs. low)’, while ‘mother ID’ and ‘block(room)’ were included as random effects. Significant test statistics are in bold; test statistics in parentheses refer to non-significant interactions that were removed from the final model.
