# Supplementary material for: Sex-specific local life-history adaptation in surface- and cave-dwelling Atlantic mollies (Poecilia mexicana)
Source: Sci Rep. 2016 Mar 10;6:22968. doi: 10.1038/srep22968 (PMC4785371; doi:10.1038/srep22968)
Supplement: Supplementary Information [file srep22968-s1.doc]

**Supplementary Material to:**

**Sex-specific local life-history adaptation in surface- and cave-dwelling Atlantic mollies (*Poecilia mexicana*)**

**Rüdiger Riesch, David N. Reznick, Martin Plath & Ingo Schlupp**

**Supplementary Material and Methods**

**Repeated measures analyses: Reproductive bout-specific changes in fecundity and interbrood interval.** We used univariate mixed-model repeated measures ANOVAs to investigate differences in fecundity (three levels, 1^st^ vs. 2^nd^ vs. 3^rd^ parturition) and interbrood interval (two levels, 1^st^ vs. 2^nd^ interbrood interval) between ecotypes and experimental treatments. Again, one set of models was run only for females from all three populations raised in the light room (i.e., under a light-dark cycle), while another was run only on cave molly females raised in different light treatments to account for the surface molly females having failed to reproduce in darkness. Light room-only models included the appropriate repeated measurement (rm; see above) as well as the factors ‘ecotype’ and ‘food regime’, and all possible two-way interactions. Cave molly-only models included the appropriate rm (see above) and the factors ‘light regime’ and ‘food regime’, and all possible two-way interactions. Both sets of models further included ‘mother ID’ and ‘block’ (for the analysis of fish raised in light) or ‘block(room)’ (for the cave molly-only analysis) as random effects. For significant model terms we again present estimated marginal means from simplified analyses as described before.

**Supplementary Results**

**Repeated measures analyses: Reproductive bout-specific changes in fecundity and interbrood interval.** Fecundity of females raised in the light room differed significantly between reproductive bouts (rm; *χ*^2^ = 6.997, df = 1, *P* = 0.008) and ecotypes (*χ*^2^ = 12.386, df = 1, *P* = 0.0004), and there was a significant interaction of ‘reproductive bout-by-ecotype’ (*χ*^2^ = 4.061, df = 1, *P* = 0.044), while food regime (*χ*^2^ = 0.507, df = 1, *P* = 0.48) had no effect. All other interactions were removed from the final model because they were not significant (*P* > 0.26 in both cases). Cave mollies produced much smaller clutches than surface mollies (at SL = 29.51 mm, cave: 3.13 ± 0.67, surface: 5.77 ± 0.63), and fecundity generally increased with each consecutive reproductive bout (at SL = 29.51 mm, 1^st^: 2.95 ± 0.35, 2^nd^: 4.18 ± 0.42, 3^rd^: 5.82 ± 0.79), but this increase was stronger in surface compared to cave mollies (Figure 3C).

In the cave molly-only analysis (females in light-dark versus dark treatments) there was only an effect of reproductive bout-by-food regime (*χ*^2^ = 3.989, df =1, *P* = 0.046), while reproductive bout itself (*χ*^2^ = 0.495, df = 1, *P* = 0.48), light regime (*χ*^2^ = 0.014, df = 1, *P* = 0.90), food regime (*χ*^2^ = 0.641, df = 1, *P* = 0.42), and all other interactions (*P* > 0.45 in both cases) were not significant. The significant interaction was due to cave mollies in the low-food treatment starting out with a lower fecundity that increased more strongly through the 2^nd^ and 3^rd^ reproductive bout (Figure 3D).

Interbrood intervals of females raised in the light room did not differ between reproductive bouts (rm: *χ*^2^ = 2.191, df = 1, *P* = 0.14), and were neither affected by the factor ecotype (*χ*^2^ = 1.183, df = 1, *P* = 0.28) nor food regime (*χ*^2^ = 0.033, df = 1, *P* = 0.86), or any of the interactions (*P* > 0.20 in all cases).

Interbrood intervals of cave mollies, however, were affected by light regime (*χ*^2^ = 7.898, df = 1, *P* = 0.005), while all other factors and interactions were not significant (rm: *χ*^2^ = 1.183, df = 1, *P* = 0.28; food regime: *χ*^2^ = 0.582, df = 1, *P* = 0.45; all interactions *P* > 0.13). This effect of light regime was significant because cave molly females in the dark room had shorter interbrood intervals than their counterparts from the light room (dark: 37.50 ± 5.82 days, light: 51.01 ± 3.56 days).

**Supplementary Discussion**

**Critical examination of the experimental protocol.** All males that matured in this experiment were considerably smaller than mature males caught in the field from the same populations in 2007 and 2008 (difference of approximately 7-8 mm for both ecotypes; compare Riesch et al. 2011*a*). For females, size at maturity in our experiment was also considerably smaller than the minimum size of reproducing females derived from field collections (cave mollies from CdA chamber V, 2007: 31 mm, 2010: 32 mm; surface mollies from Río Amatán, 2007: 30 mm, from Arroyo Bonita, 2008: 33 mm; Riesch et al. 2010*b*). Two not mutually exclusive mechanisms might explain these patterns. First, growth conditions in the laboratory might not be as favorable on average as in the field. Second, all fish were raised in social isolation (except for short-term pairing of females with males to ensure fertilization), and so larger males and females in nature may represent a genotype-by-environment interaction or a purely environmental effect, with the social environment potentially playing an important role (e.g., *Xiphophorus variatus*: Borowsky 1973; *Phallichthys quadripunctatus*: Kolluru and Reznick 1996).

Furthermore, the importance of environmental cues in early life cannot be ignored (developmental plasticity: Horton 2005; West-Eberhard 2005). In our experiment, we exposed neonates to benign conditions for the first 37 days of their life, and only then exposed them to the different experimental treatments. This was necessary to prevent early life mortality as poeciliid neonates do not respond well to handling and stress during the first few days of their lives (all authors, personal observation). However, this is also likely to have biased some of our results by potentially having early-life environmental cues result in slightly more convergent phenotypes between cave- and surface mollies than would have been the case had we exposed neonates immediately to the different experimental treatments.

**Supplementary Table S1.** Descriptive statistics (mean±s.d.) for life-history traits at maturity of first generation laboratory-born male *Poecilia mexicana*.

| Ecotype | Light regime | Food regime | *N^a^* | SL [mm] | Age [d] | Maturation time [d] | Growth rate  [mm/d] | Male lean  weight^b^ [g] | Male fat content [%] | GSI^c^ [%] |
| --- | --- | --- | --- | --- | --- | --- | --- | --- | --- | --- |
| Cave | Light-dark | High | 5/5 | 24.0±1.2 | 109.80±9.15 | 40.40±6.19 | 0.065±0.047 | 0.04±0.01 | 9.97±0.04 | 0.89±0.01 |
|  |  | Low | 6/6 | 21.8±1.8 | 158.50±49.00 | 48.67±3.98 | 0.040±0.022 | 0.05±0.01 | 1.76±0.04 | 1.03±0.01 |
|  | Dark | High | 8/8 | 22.6±0.9 | 130.50±35.69 | 37.12±4.09 | 0.039±0.017 | 0.05±0.01 | 5.36±0.04 | 1.11±0.01 |
|  |  | Low | 8/8 | 21.3±1.8 | 185.88±50.51 | 35.50±4.69 | 0.033±0.009 | 0.04±0.01 | 3.30±0.04 | 2.05±0.01 |
| Surface | Light-dark | High | 11/11 | 23.2±1.4 | 102.64±12.82 | 34.91±3.70 | 0.083±0.047 | 0.05±0.01 | 14.29±0.04 | 0.89±0.01 |
|  |  | Low | 7/7 | 20.9±1.6 | 118.86±40.65 | 41.14±5.46 | 0.068±0.019 | 0.05±0.01 | 4.20±0.04 | 1.21±0.01 |
|  | Dark | High | 12/12 | 20.7±1.3 | 100.42±19.59 | 33.58±4.96 | 0.083±0.023 | 0.05±0.01 | 7.56±0.04 | 1.14±0.01 |
|  |  | Low | 7/7 | 20.0±2.2 | 182.71±78.78 | 38.57±8.73 | 0.053±0.029 | 0.05±0.01 | 3.44±0.04 | 2.13±0.01 |

^a^ numerator corresponds to males that successfully reached sexual maturity; denominator equals the total number of males in the experiment

^b^ estimated marginal means±s.d. from a GLM with SL as a covariate.

^c^ GSI (=gonadosomatic index): Proportion of total dry weight which consists of testes.

**Supplementary Table S2.** Descriptive statistics (mean±s.d.) for life–history traits of first-generation laboratory-born female *Poecilia mexicana*.

| Ecotype | Light regime | Food regime | *N*^a^ | SL at 1^st^ parturition [mm] | Age at 1^st^ parturition [d] | SL at 3^rd^ parturition [mm] | Female fat  Content^b^ [%] | Female lean weight^b^ [g] | Fecundity^b^  [# offspring] | RA^b,c^ [%] |
| --- | --- | --- | --- | --- | --- | --- | --- | --- | --- | --- |
| Cave | Light-dark | High | 10/10 | 30.0±3.6 | 220.40±54.59 | 34.7±2.8 | 5.53±2.85 | 0.13±0.03 | 3.48±1.80 | 19.62±6.01 |
|  |  | Low | 10/11 | 27.1±1.5 | 283.40±22.24 | 30.4±2.8 | 2.58±2.85 | 0.12±0.03 | 2.84±1.86 | 15.57±6.32 |
|  | Dark | High | 8/10 | 29.4±2.7 | 250.38±49.60 | 32.4±2.3 | 4.19±2.83 | 0.15±0.03 | 3.14±1.69 | 12.79±5.66 |
|  |  | Low | 3/7 | 26.3±1.2 | 343.67±15.57 | 31.0±0.0 | 0.30±3.29 | 0.15±0.04 | 3.13±2.01 | 6.44±6.75 |
| Surface | Light-dark | High | 10/11 | 32.7±2.4 | 243.80±41.43 | 37.0±3.4 | 5.31±3.48 | 0.24±0.04 | 6.40±2.13 | 11.98±7.27 |
|  |  | Low | 6/12 | 27.7±1.6 | 283.67±15.71 | 30.5±1.1 | 3.54±2.94 | 0.17±0.03 | 5.40±2.29 | 12.08±6.12 |
|  | Dark | High | 1/7 | 29.0±0.0 | 239.00±0.00 | 27.0±0.0 | 11.00±0.00 | 0.18±0.00 | 6.97±0.00 | 11.00±0.00 |
|  |  | Low | 0/9 | - | - | - | - | - | - | - |

| Ecotype | Light regime | Food regime | Interbrood interval [d] | Neonate SL^b^ [mm] | Neonate dry weight^b^ [mg] | Neonate fat content^b^ [%] |
| --- | --- | --- | --- | --- | --- | --- |
| Cave | Light-dark | High | 52.95±17.07 | 10.17±0.42 | 6.47±0.65 | 6.12±1.60 |
|  |  | Low | 47.35±3.45 | 10.55±0.38 | 6.61±1.14 | 5.57±1.14 |
|  | Dark | High | 40.13±7.30 | 10.05±0.53 | 6.55±1.59 | 5.35±1.20 |
|  |  | Low | 37.83±6.25 | 9.77±0.25 | 5.77±0.67 | 6.22±1.23 |
| Surface | Light-dark | High | 44.40±8.57 | 8.72±0.38 | 4.15±0.59 | 6.08±1.96 |
|  |  | Low | 48.42±9.13 | 8.56±0.61 | 3.51±0.98 | 5.21±1.72 |
|  | Dark | High | 36.50±0.00 | 8.32±0.00 | 3.35±0.00 | 3.56±0.00 |
|  |  | Low | - | - | - | - |

^a^ numerator corresponds to females that successfully reproduced three times; denominator equals the total number of females in the experiment

^b^ estimated marginal means±s.d.. from a GLM with SL as a covariate.

^c^ Reproductive allocation: Proportion of total dry weight which consists of developing embryos.

**Supplementary Table S3.** Growth rates and age at sacrifice of female *P. mexicana* that either failed to reproduce within their first year of life, contracted columnaris disease, or that only reproduced once or twice and then ceased reproduction. SL and wet weight were measured every two weeks, beginning in week 5.

| Ecotype | Light regime | Food regime | Reason for sacrifice | Growth rate [mm/d, mg/d] | Age at sacrifice [d] | Comments |
| --- | --- | --- | --- | --- | --- | --- |
| Surface | Dark | Low | Columnaris disease | 0.034, 0.639 | 300 | Continuous SL and wet weight increase |
| Surface | Dark | High | Columnaris disease | 0.061, 2.701 | 320 | Continuous SL and wet weight increase |
| Surface | Dark | Low | Columnaris disease | 0.026, 0.594 | 346 | SL and wet weight decreased on the last measurement prior to sacrifice |
| Surface | Dark | High | Columnaris disease | 0.050, 1.929 | 320 | Wet weight decreased on the last measurement prior to sacrifice |
| Surface | Dark | High | Columnaris disease | 0.043, 2.422 | 361 | Continuous SL and wet weight increase |
| Surface | Dark | Low | Columnaris disease | 0.032, 0.942 | 358 | Continuous SL and wet weight increase |
| Surface | Dark | High | Columnaris disease | 0.047, 1.677 | 360 | Continuous SL and wet weight increase |
| Surface | Dark | Low | 1 year in experiment | 0.024, 0.685 | 365 | Wet weight decreased on the last measurement prior to sacrifice |
| Surface | Light-dark | Low | 1 year in experiment | 0.047, 1.770 | 365 | Continuous SL and wet weight increase |
| Surface | Dark | Low | 1 year in experiment | 0.034, 0.901 | 365 | Wet weight decreased on the last measurement prior to sacrifice |
| Surface | Dark | High | 1 year in experiment | 0.051, 2.470 | 365 | Continuous SL and wet weight increase |
| Surface | Light-dark | Low | Columnaris disease | 0.049, 2.105 | 314 | Continuous SL and wet weight increase |
| Surface | Dark | Low | 1 year in experiment | 0.034, 1.056 | 365 | Continuous SL and wet weight increase |
| Surface | Light-dark | Low | 1 year in experiment | 0.056, 2.329 | 365 | Continuous SL and wet weight increase |
| Surface | Dark | Low | 1 year in experiment | 0.043, 1.459 | 365 | Continuous SL and wet weight increase |
| Surface | Light-dark | Low | 1 year in experiment | 0.059, 2.484 | 365 | Continuous SL and wet weight increase |
| Cave | Dark | Low | 1 year in experiment | 0.031, 1.025 | 365 | Continuous SL and wet weight increase |
| Cave | Dark | Low | 1 year in experiment | 0.031, 1.025 | 365 | Continuous SL and wet weight increase |
| Cave | Dark | Low | 1 year in experiment | 0.028, 0.870 | 365 | Continuous SL and wet weight increase |
| Cave | Dark | High | Eye infection | *0.054, 2.101 | 383 | 2 successful litters, but stopped eating for several days prior to sacrifice |
| Surface | Light-dark | Low | End of experiment | *0.053, 2.218 | 502 | 1 successful litter but then ceased reproduction |
| Surface | Light-dark | Low | Columnaris disease | *0.051, 1.973 | 406 | 1 successful litter, but then contracted columnaris disease |

* pre-maturation growth rates
